# Efficient frequency generation in phoXonic cavities based on hollow whispering gallery mode resonators

**DOI:** 10.1038/srep44198

**Published:** 2017-03-07

**Authors:** Daniele Farnesi, Giancarlo Righini, Gualtiero Nunzi Conti, Silvia Soria

**Affiliations:** 1 CNR-IFAC- Institute of Applied Physics, Sesto Fiorentino, 50019, Italy; 2 Centro Studi e Ricerche “E. Fermi”, Rome, 00184, Italy

## Abstract

We report on nonlinear optical effects on phoxonic cavities based on hollow whispering gallery mode resonators pumped with a continuous wave laser. We observed stimulated scattering effects such as Brillouin and Raman, Kerr effects such as degenerated and non-degenerated four wave mixing, and dispersive wave generation. These effects happened concomitantly. Hollow resonators give rise to a very rich nonlinear scenario due to the coexistence of several family modes.

Stimulated Brillouin Scattering (SBS) in devices that highly confine light has attracted much attention in the last years^1^,^2^,^3^,^4^. Among these devices, whispering gallery mode resonators (WGMR) have shown to be an excellent enhancement platform to study light matter interactions such as stimulated nonlinear optical processes and frequency generation. WGMR can confine light in very small volumes and posses ultra high Q-factors^5^,^6^. The high photon density and their long lifetime ensure an efficient nonlinear light-matter interaction and give the possibility to study, from the fundamental point of view, Kerr interactions and stimulated phenomena like Raman and Brillouin^4^,^7^,^8^,^9^,^10^.

SBS is an inelastic scattering process that results from the coherent interaction of light photons and acoustic phonons. The photon-phonon interaction is enhanced due to the overlap of both waves inside the WGMR, which acts as a dual photonic-phononic or phoxonic cavity^11^,^12^. SBS, like stimulated Raman scattering (SRS), is a pure gain process and therefore, is automatically phase-matched. SBS has one of the largest gain coefficients but a small gain bandwidth^13^. The narrow bandwidth would require that the Brillouin frequency shift equals the free spectral range (FSR) of the WGMR, placing stringent conditions on WGMR geometries^14^,^15^,^16^. However, because of their eccentricity, in microbubble resonators this condition can be bypassed by using high order modes^17^,^18^ with vertical FSR smaller than the fundamental FSR^10^,^19^,^20^.

WGMR have been fabricated in a large variety of geometries and materials^21^. One of the latest geometries, the microbubble^22^ or microbottle^23^ has attracted much attention lately. Another advantage of the hollow structure of the bubble is that it allows flowing liquids or gas inside the WGMR, giving the possibility of tailoring the dispersion^24^ or resonant properties^25^,^26^. The majority of applications of microbubble resonators (MBR) are in the sensing field: refractometers^27^,^28^, mass sensors^29^, temperature^30^, pressure^31^ and viscosity^32^. More recently, MBR have been used for fundamental studies in nonlinear optics^20^,^26^,^33^ and lasing^34^. In refs 26 and 33, the MBR used have very thick walls (of about 10–15 μm) compared to ours (2–4 μm), and the SBS and FWM observed in ref. 26 are not either simultaneous or the SBS line does not act as a pump in SBS-uncoupled FWM processes. Regarding the efficiency of the SBS lines, we are presenting results that are at least more than one order of magnitude higher. Our objective here is to demonstrate that MBR with thin walls are more efficient for SBS excitation and show a richer nonlinear scenario than other on-chip WGMR.

Summarizing, we experimentally show that MBR of large diameters and thin walls (2 μm) can enhance the Brillouin lasing efficiency, cascading up to the 4^th^ order in both forward and backward directions. Even and odd orders are observed in both directions, but showing different lasing efficiency (even (odd) orders are more efficient in forward (backward) direction). We also report on degenerated four wave mixing (FWM) from the Brillouin laser, non-degenerated FWM, dispersive waves and SBS and SRS appearing simultaneously. Clear advantages of MBR is that their modal structure allow satisfying the different phase matching and the multi-resonant conditions required by the above mentioned NLO effects, firstly; and the hollow structure also consents to tailor dispersion or resonant conditions easily.

## Experimental set-up and physical principles

**Figure 1:**
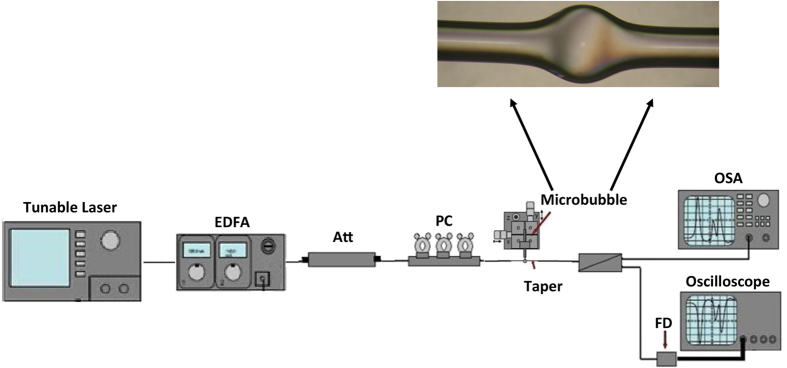
Experimental set-up.

The experimental setup is shown in Fig. 1. The laser light from a tunable diode laser (TDL) is amplified with an erbium-doped fiber amplifier (EDFA, IPG) and after passing an attenuator (ATT) and polarization controller (POL) is coupled to the equator of a MBR by means of a tapered fiber, produced in-house too. The laser is tuned into a resonance from high to low frequencies, which results in thermal self-locking^35^ of the WGMR mode to the pump laser. A splitter at the end of the taper sends a part of the signal into an optical spectrum analyzer (OSA, ANDO AQ6317B) and part to a photo-detector and oscilloscope (TEKTRONIX). SBS, SRS and FWM were detected on an OSA in forward and backward direction by using a circulator, directly transmitted by the taper fiber. A fiber optical circulator (CIRC) was inserted before the taper in order to extract the feedback signal and observe forward and also in backward direction of lasing. The maximum launched pump power is about 200 mW. We performed the experiments at room temperature and atmospheric pressure.

SBS corresponds to a lattice oscillation that can be described as ω_p_ = ω_s_ + Ω_B_, a pump photon is scattered into a Stokes photon and an acoustical phonon at the frequency Ω_B_. Light scattering can occur in both directions, forward with frequencies ranging the MHz-GHz range; and backward with frequencies in the GHz range^9^. The SBS frequency scales with the optical one and it is about 11 GHz in silica glass, with a bandwidth ranging 20 to 60 MHz at telecom frequencies. In our experiments, the free spectral range (FSR) of our MBR is 140 GHz (diameter about 475 μm) and 100 GHz (diameter about 675 μm). Therefore, we can obtain SBS only by using mode families with the same azimuthal but different vertical quantum number (their degeneracy is effectively removed because of the strong eccentricity of the bubble), whose FSR is much less than the FSR of the azimuthal number^20^. At elevated pump power, cascaded SBS can happen, generating high-order Stokes lines at ω_ns_ = ω_p_ − nΩ_B_. The phoxonic MBR supports at least three resonances, two optical –pump and Stokes- and one acoustical, which are highly overlapping.

**Figure 2:**
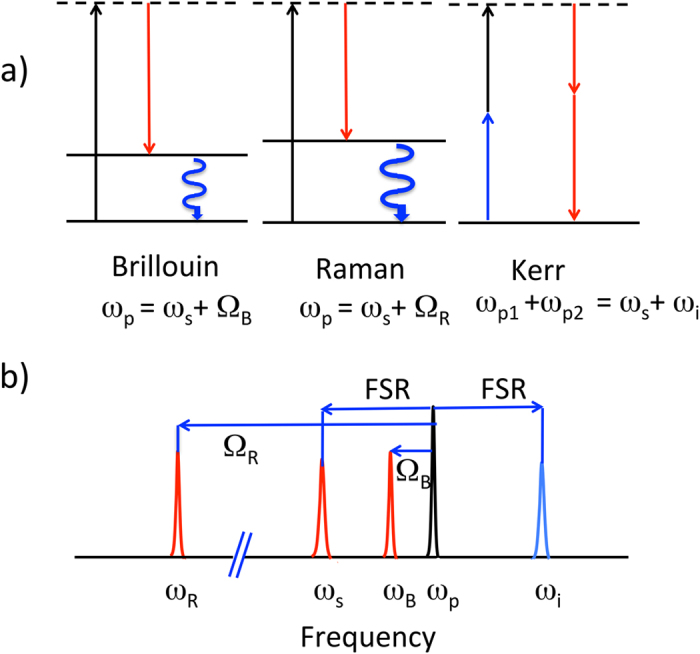
(**a**) Sketch of the simplified energy levels for Brillouin, Raman and Kerr scattering. The dashed lines represent the virtual energy states. (**b**) Illustration of the multiresonant frequency conversion for Raman, Brillouin and Kerr. FSR: free spectral range. SBS and SRS process generate only Stokes fields separated from the pump by Ω_B_ (Brillouin) or Ω_R_ (Raman). FWM generates Stokes and anti-Stokes fields separated by the pump multiples of FSR. Stokes fields are red, anti-Stokes are blue.

SRS corresponds to a molecular vibration transition that can be described as ω_p_ = ω_s_ + Ω_R_, a pump photon is scattered to a Stokes photon and an optical phonon at the frequency Ω_R_. The SRS frequency scales with the optical one and it is about 10 THz in silica glass, with a bandwidth ranging several THz at telecom frequencies. The Kerr effect is due to an electron cloud oscillation, quasi instantaneous, that can be describe by FWM (hyper-parametrical oscillations). FWM can be degenerated 2ω_p_ = ω_s_ + ω_i_ or not ω_p1_ + ω_p2_ = ω_s_ + ω_i_. FWM is coherent and obeys precise phase matching conditions. In WGMR, the phase matching condition is related to the momentum conservation in the azimuthal mode indices: the frequency spacing matches single or multiple FSR. The presence of anti-Stokes frequencies is a clear indication of a hyper-parametrical oscillation^13^,^20^,^36^ since SBS and SRS cannot generate anti-Stokes fields. Figure 2 shows an schematic illustration of the energy levels for Brillouin, Raman and Kerr scattering together with an schematic drawing of the multiresonant optical frequency conversion.

## Results

**Figure 3:**
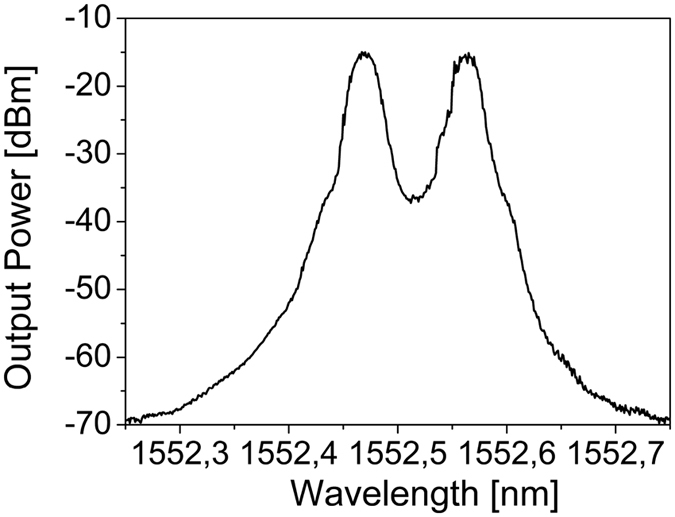
First order SBS Stokes lines in backward direction for a MBR of diameter about 475 μm and wall thickness of about 3–4 μm.

Figure 3 shows the optical spectrum corresponding to the 475 μm diameter MBR for a launched power of 80 mW, in backward direction for a pump wavelength of 1552,47 nm and a SBS Stokes line shifted of 0,09 nm (11,2 GHz).

**Figure 4:**
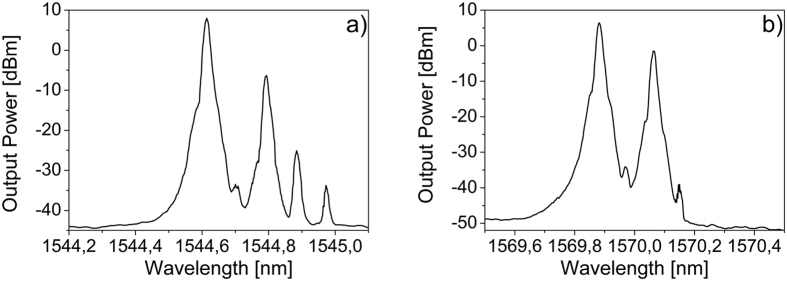
Cascaded forward SBS in a MBR of diameter about 675 μm and wall thickness of about 2 μm: (**a**) for a λp = 1544,614 nm up to the 4^th^ order; and (**b**) for λp = 1569,882 nm up to the 3^rd^ order but with 17% of efficiency in the 2^nd^ order. The launched pump power is 200 mW.

Increasing the launched pump power up to 200 mW, we have obtained cascaded SBS up to the 4^th^ order in a MBR of diameter about 675 μm and wall thickness of about 2 μm. As expected, the Stokes lines are shifted by 11, 22, 33 and 44 GHz for a pump wavelength centered at 1544,614 nm (Fig. 4a). By changing the pump wavelength at 1569,882 nm, we could observe cascaded SBS up to the 3^rd^ order only, but with a high efficiency in the 2^nd^ order SBS lasing. In this case, the efficiency is up to 17%.

**Figure 5:**
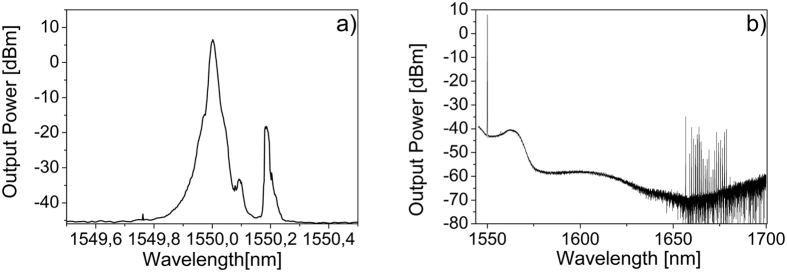
SBS and SRS spectra in forward direction from a MBR of about 675 μm of diameter at a pump wavelength of 1550 nm: (**a**) cascaded SBS up to the 2^nd^ order and a low efficiency anti-Stokes line and (**b**) SRS family modes.

SRS was also observed contemporaneously to SBS at the same launched pump power of about 200 mW. Figure 5 shows SBS and SRS happening simultaneously at 1550 nm, in forward direction. The Raman Stokes line is separated by 13 THz (110 nm) from the pump, as expected for a wavelength centered at 1550 nm.

**Figure 6:**
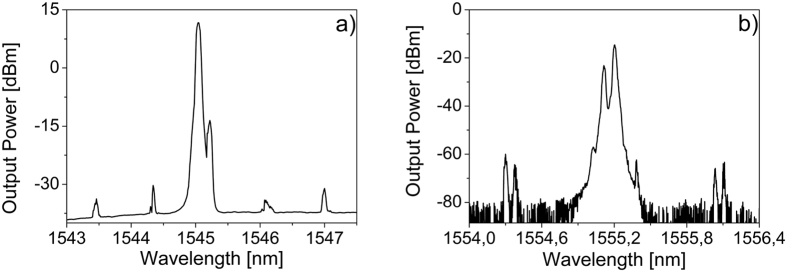
SBS and degenerated FWM in: (**a**) forward direction (wavelength pump 1545,04 nm) and (**b**) backward direction (wavelength pump 1555,026 nm).

Degenerated FWM from the stimulated Brillouin laser line is also possible in both forward and backward direction. In forward direction, we observed cascaded FWM from the second order Brillouin laser line (1545,22 nm) for a pump wavelength centered at 1545,04 nm. The FWM lines are separated by a FSR (Fig. 6a). In backward direction, we observed two sets of Stokes and anti-Stokes lines very close to each other. The first set is separated by one FSR from the 2^nd^ order stimulated Brillouin laser line, the second set is separated by a non-integer multiple of the FSR (Fig. 6b).

**Figure 7:**
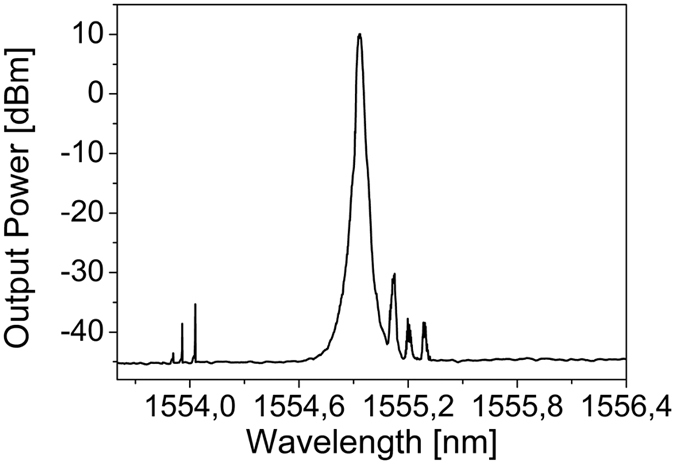
SBS and dispersive waves in forward direction (wavelength pump 1554,94 nm).

We also observed cascaded SBS together with some anti-Stokes components that could correspond to dispersive wave multiplets more than non-degenerated FWM either between the pump and the Raman Stokes or between the Stokes components (Fig. 7).

## Discussion

In guiding systems showing translationally invariance, SBS can happen in forward direction (FSBS), were both pump and scattered waves are co-directional and coupled through transverse standing wave phonons; and in backward direction (BSBS), were the waves are counter-propagating and coupled through traveling wave phonons. FSBS processes are usually very weak unless there is a lateral phonon confinement^3^,^37^,^38^ and even though, they are weaker than BSBS due to superior confinement of longitudinal phonons^3^. However, giant enhancement of both processes has been theoretically proven when taking into account the contributions of radiation pressure or boundary-induced nonlinearities^3^,^39^.

WGMR, like nanoscale waveguides^3^,^38^, microstructured optical fibers^37^ and suspended silicon waveguides^40^ can efficiently generate FSBS, revealing cascaded Brillouin interactions with characteristics similar to Raman lasing^7^,^8^. In our case, we have observed cascaded FSBS up to the forth order showing even and odd orders, due to the presence of Rayleigh scattering in the resonator, (see Fig. 4) with high efficiency as expected for thin shells^39^. It is worth to note, that even though we have observed cascaded SBS, we have not observed the correspondent stimulated anti-Stokes Brillouin scattering differently to SRS where stimulated anti-Stokes Raman scattering (SARS) can also occur^36^,^41^.

We have also observed SRS and FWM occurring simultaneously with SBS. The observed FWM is both degenerated and non-degenerated and it arises from the strong 2^nd^ order Brillouin laser line in forward and backward direction. Signal and idler are separated by an integer multiple of the FSR, indicating intermodal interactions.

The spatial mode interaction within a microresonator is already a demonstrated mechanism for dispersive wave generation, the optical analog of Cherenkov radiation^42^,^43^,^44^. Dispersive wave generation can be, thus, linked to cascaded FWM^42^ when high-order dispersion is present in the resonator and due to linearly interacting families of equidistant modes with slightly different FSR^43^,^44^. In MBR coexist several family modes, giving rise to a very rich nonlinear scenario.

## Conclusions

In conclusion, we reported on the simultaneous excitation of Brillouin, Raman and Kerr effects in silica MBR. We showed that phoxonic MBRs can act as enhancement platforms for multiresonant nonlinear phenomena, while keeping the dimensions in the micrometer scale. The coexistence of several family modes in the same cavity favors the fulfillment of the different frequency and phase matching conditions, co-exciting different nonlinear effects. The denser spectra and the capacity of tailoring the dispersion make MBR a very attractive class of WGMR that can be used not only in fundamental studies such as nonlinear optics but also in practical ones such as sensing.

## Methods

The MBRs were fabricated from slightly pressurized silica capillaries using a modified fusion splicer, where the electrodes could rotate by 360°, in order to obtain uniform heating of the capillary. The detailed fabrication procedure can be found in ref. 27. Figure 1 shows an optical image of a MBR fabricated with this method, which was created from a capillary with an ID of 200 μm OD of about 280 μm (Postnova Z-FSS-200280 capillary). The diameters of the microbubbles used in these experiments range from a minimum diameter of about 475 μm up to a maximum diameter of about 640 μm with wall thicknesses are ranging from 3 μm ± 0.5 μm and 2 μm ± 0.5 μm, respectively. The corresponding quality factors Q are about 3.5 10^7^.

## Additional Information

**How to cite this article:** Farnesi, D. *et al*. Efficient frequency generation in phoXonic cavities based on hollow whispering gallery mode resonators. *Sci. Rep.* **7**, 44198; doi: 10.1038/srep44198 (2017).

**Publisher's note:** Springer Nature remains neutral with regard to jurisdictional claims in published maps and institutional affiliations.

## References

[b1] Ippen E. P. & Stolen R. H. Stimulated Brillouin scattering in optical fibers. App. Phys. Lett. 21, 539–541 (1972).

[b2] Pant R. . Cavity enhanced stimulated Brillouin scattering in an optical chip for multiorder Stokes generation. Opt. Lett. 36, 3687–3689 (2011). 21931433 10.1364/OL.36.003687

[b3] Rakich P. T., Reinke C., Camacho R., Davids P. & Wang Z. Giant enhancement of stimulated Brillouin scattering in subwavelength limit. Phys. Rev. X 2, 011008 (2012).

[b4] Grudinin I. S., Matsko A. B. & Maleki L. Brillouin lasing with a CaF2 whispering gallery mode resonator. Phys. Rev. Lett. 102, 043902 (2009). 19257418 10.1103/PhysRevLett.102.043902

[b5] Savchenkov A. A., MAtsko A. B., Ilchenko V. S. & Maleki L. Optical resonator with ten million finesse. Opt. Express 15, 6768–6773 (2007). 19546987 10.1364/oe.15.006768

[b6] Vahala K. J. Optical Microcavities. Nature 424, 839–846 (2003). 12917698 10.1038/nature01939

[b7] Spillane S. M., Kippenberg T. J. & Vahala K. J. Ultralow threshold Raman laser using a spherical dielectric microcavity. Nature 415, 621–623 (2002). 11832940 10.1038/415621a

[b8] Kippenberg T. J., Spillane S. M. & Vahala K. J. Kerr nonlinearity optical parametric oscillation in an ultrahigh Q toroid microcavity. Phys. Rev. Lett. 93, 083904 (2004). 15447188 10.1103/PhysRevLett.93.083904

[b9] Bahl G., Tomes M., Marquardt F. & Carmon T. Observation of spontaneous Brillouin cooling. Nat. Phys. 8, 203–207 (2012).

[b10] Lin G., Diallo S., Dudley J. M. & Chembo Y. K. Universal nonlinear scattering in ultra-high Q whispering gallery mode resonators. Opt. Express 24, 14880–14894 (2016). 27410640 10.1364/OE.24.014880

[b11] Maldovan M. & Thomas E. L. Simultaneous localization of photons and phonons in two-dimensional periodic structures. Appl. Phys. Lett. 88, 251907 (2006).

[b12] Rolland Q. . Acousto-optic couplings in two-dimensional phoxonic crystal cavities. Appl. Phys. Lett. 101, 061109 (2012).

[b13] Boyd R. W. Nonlinear Optics 2^nd^ edn (Academic, San Diego, 2003).

[b14] Lee H. . Chemically etched ultrahigh Q resonator on a silicon chip. Nat. Photonics 6, 369–373 (2012).

[b15] Eggleton B. J., Poulton Ch. G. & Pant R. Inducing and harnessing stimulated Brillouin scattering in photonic integrated circuits. Adv. Opt. Photon. 5, 536–587 (2013).

[b16] Asano M. . Stimulated Brillouin scattering and Brillouin coupled four wave mixing in a silica microbottle resonator. Opt. Express 24, 12082–12092 (2016). 27410129 10.1364/OE.24.012082

[b17] Lin G. . Cascaded Brillouin lasing in monolithic barium fluoride whispering gallery mode resonators. Appl. Phys. Lett. 105, 231103 (2014).

[b18] Guo C. . Ultralow-threshold cascaded Brillouin microlaser for tunable microwave generation. Opt. Lett. 40, 4971–4973 (2015). 26512496 10.1364/OL.40.004971

[b19] Liang W. . Miniature multioctave light source based on a monolithic microcavity. Optica 2, 40–47 (2015).

[b20] Farnesi D., Barucci A., Righini G. C., Nunzi Conti G. & Soria S. Generation of hyper-parametric oscillations in silica microbubbles. Opt. Lett. 40, 4508–4511 (2015). 26421568 10.1364/OL.40.004508

[b21] Chiasera A. . Spherical whispering-gallery-mode microresonators. Laser Photon. Rev. 4, 457–483 (2010).

[b22] Sumetsky M. Whispering gallery bottle microcavities: the three dimensional etalon. Opt. Lett. 29, 8–10 (2004). 14719643 10.1364/ol.29.000008

[b23] Murugan G. S., Petrovich N. M., Jung Y., Wilkinson J. S. & Zervas M. N. Hollow bottle optical microresonators. Opt. Express 19, 20773–20784 (2011). 21997087 10.1364/OE.19.020773

[b24] Riesen N., Zhang W. Q. & Monro T. Dispersion in silica microbubbles. Opt. Lett. 41, 1257–1260 (2016). 26977683 10.1364/OL.41.001257

[b25] Henze R., Seifert T., Ward J. & Benson O. Tuning whispering gallery modes using internal aerostatic pressure. Opt. Lett. 36 4536–4538 (2011). 22139234 10.1364/OL.36.004536

[b26] Lu Q., Liu S., Wu X., Liu L. & Xu L. Stimulated Brilluoin laser and frequency comb generation in high-Q microbubble resonators. Opt. Lett. 41, 1736–1739 (2016). 27082332 10.1364/OL.41.001736

[b27] Berneschi S. . High Q silica microbubble resonators fabricated by arc discharge. Opt. Lett. 36, 3521–3522 (2011). 21886264 10.1364/OL.36.003521

[b28] Li M., Wu X., Liu L., Fan X. & Xu L. Self-referencing optofluidic ring resonator sensor for highly sensitive biomolecular detection. Anal. Chem. 85, 9328–9332 (2013). 23992426 10.1021/ac402174x

[b29] Kim K. H. & Fan X. Surface sensitive microfluidic optomechanical ring resonator sensors. Appl. Phys. Lett. 105, 191101 (2014).

[b30] Ward J. M., Yang Y. & Nic Chormaic S. Highly Sensitive Temperature Measurements With Liquid-Core Microbubble Resonators. IEEE Photon. Tech. Lett. 25, 2350 (2013).

[b31] Yang Y., Ward J. & Nic Chormaic S. Quasi-droplet microbubbles for high resolution sensing applications Opt. Express 22, 6881 (2014). 24664037 10.1364/OE.22.006881

[b32] Han K., Zhu K. & Bahl G. Opto-mechano-fluidic viscometer. Appl. Phys. Lett. 105, 014103 (2014).

[b33] Bahl G. . Brillouin cavity optomechanics with microfluidic devices. Nature Commun. 4, 1994 (2013). 23744103 10.1038/ncomms2994

[b34] Ward J. M., Yang Y. & Nic Chormaic S. Glass-on-glass fabrication of bottle-shaped tunable micro-lasers and their applications, Sci. Rep. 6, 25152 (2016). 27121151 10.1038/srep25152 PMC4848646

[b35] Carmon T., Yang L. & Vahala K. Dynamical thermal behavior and thermal self-stability of microcavities. Opt. Express 12, 4742 (2004). 19484026 10.1364/opex.12.004742

[b36] Farnesi D. . Stimulated Anti-Stokes Raman Scattering in silica microspheres. Opt. Lett. 39, 5993–5996 (2014). 25361138 10.1364/OL.39.005993

[b37] Kang M. S., Nazarkin A., Brenn A. & Russell P. S. J. Tightly trapped acoustic phonons in photonic crystal fibers as highly nonlinear artificial raman oscillators. Nat. Phys. 5, 276–280 (2009).

[b38] Qiu W. . Stimulated Brillouin scattering in nanoscale silicon step-index waveguides: a general framework of selection rules and calculating SBS gain. Opt. Express 21, 31402–31419 (2013). 24514715 10.1364/OE.21.031402

[b39] Dostart N., Kim S. & Bahl G.. Giant Gain Enhancement in Surface-Confined Resonant Stimulated Brillouin Scattering. Laser Photonics Rev. 9(6), 689–705 (2015).

[b40] Wolff C., Steel M. J., Eggleton B. J. & Poulton C. G. Acoustic build-up in on-chip stimulated Brillouin scattering. Sci. Rep. 5, 13656 (2015). 26338720 10.1038/srep13656 PMC4559895

[b41] Leach D. H., Chang R. K. & Acker W. P. Stimulated anti-Stokes Raman scattering in microdroplets. Opt. Lett. 17, 387–389 (1992). 19784336 10.1364/ol.17.000387

[b42] Erkintalo M., Xu Y. Q., Murdoch S. G., Dudley J. M. & Genty G. Cascaded Phase Matching and Nonlinear Symmetry Breaking in Fiber Frequency Combs. Phys. Rev. Lett. 109, 223904 (2012). 23368122 10.1103/PhysRevLett.109.223904

[b43] Matsko A. B., Lang W., Savchenkov A. A., Eliyahu D. & Maleki L. Optical Cherenkov radiation in overmoded microresonators. Opt. Lett. 41, 2907–2910 (2016). 27367062 10.1364/OL.41.002907

[b44] Yang Q.-F., Yi X., Yang K. Y. & Vahala K. Spatial-mode-interaction-induced dispersive-waves and their active tuning in microresonators. Optica 3, 1132–1135 (2016).

